# Sticky Gutta-Percha Fillings

**Published:** 1900-11-15

**Authors:** J. F. P. Hodgson


Sticky Gutta-Percha Fillings.—Touch warmed gutta-percha, on its way to the cavity, with oil of cajeput. On account of the increased stickiness of the gutta percha so treated, the filling actually cements itself to the walls of the cavity. It can even be applied wet, and so is of real value in treating a patient ill in bed, etc.—*J. F. P. Hodgson, International Dental Journal.*

